# Pharmacological Mechanisms Underlying the Neuroprotective Effects of *Alpinia oxyphylla* Miq. on Alzheimer’s Disease

**DOI:** 10.3390/ijms21062071

**Published:** 2020-03-18

**Authors:** Jia Xu, Fang Wang, Jiejie Guo, Chunshuang Xu, Yanzi Cao, Zhiling Fang, Qinwen Wang

**Affiliations:** Ningbo Key Laboratory of Behavioral Neuroscience, Zhejiang Provincial Key Laboratory of Pathophysiology, School of Medicine, Ningbo University, Ningbo, Zhejiang 315211, China; xujia@nbu.edu.cn (J.X.); wangf@zjpu.edu.cn (F.W.); gjj_19880608@126.com (J.G.); xuchunshuang@nbu.edu.cn (C.X.); gabrielzuo@hotmail.com (Y.C.); zhilingf0723@163.com (Z.F.)

**Keywords:** *Alpinia oxyphylla* Miq., terpenes, Alzheimer’s disease, systems pharmacology, molecular docking, neuroprotection, amyloid-beta

## Abstract

*Alpinia oxyphylla* Miq. (i.e., *A. oxyphylla*), a traditional Chinese medicine, can exert neuroprotective effects in ameliorating mild cognitive impairment and improving the pathological hallmarks of Alzheimer’s disease (AD). Here, 50 active compounds and 164 putative targets were collected and identified with 251 clinically tested AD-associated target proteins using network pharmacology approaches. Based on the Gene Ontology/Kyoto Encyclopedia of Genes and Genomes pathway enrichments, the compound-target-pathway-disease/protein–protein interaction network constructions, and the network topological analysis, we concluded that *A. oxyphylla* may have neuroprotective effects by regulating neurotransmitter function, as well as brain plasticity in neuronal networks. Moreover, closely-related AD proteins, including the amyloid-beta precursor protein, the estrogen receptor 1, acetylcholinesterase, and nitric oxide synthase 2, were selected as the bottleneck nodes of network for further verification by molecular docking. Our analytical results demonstrated that terpene, as the main compound of *A. oxyphylla* extract, exerts neuroprotective effects, providing new insights into the development of a natural therapy for the prevention and treatment of AD.

## 1. Introduction

Alzheimer’s disease (AD) is the most prevalent neurodegenerative disorder, characterized by brain atrophy, progressive cognitive dysfunction, and behavioral disturbances [1]. The cardinal neuropathological features of AD include extracellular accumulations of amyloid beta (Aβ) peptide [2], neurofibrillary tangles (NFTs) of hyper-phosphorylated tau proteins [3], neuropil threads, dystrophic neuritis [4,5], astrogliosis, microglial activation [6], and overall neurodegeneration of the brain [6,7,8]. Several hypotheses about cholinergic system dysfunction [9], Aβ deposits, oxidative stress [10], inflammatory pathways [11], calcium signaling dysfunction [12], hormone imbalance [13], and genetic components [14] have been considered to play important roles in the occurrence and development of AD, although the etiology of this disease is still not precisely known [15]. In addition, although several therapies have been used as compensation for the cholinergic neuronal loss and reduction or prevention of amyloid/tau aggregation and toxicity, such as gene therapy, vaccines, anti-inflammatory agents [16], cholesterol-lowering agents, anti-oxidants [17], and hormone therapy [18] for AD, these single targeted therapies have often been unsuccessful [19,20]. Traditional Chinese Medicine (TCM), with thousands of years of history in China, has received widespread attention, particularly given the few side effects it carries, multi-target treatments, and natural origins [21,22]. These medicines have been successfully utilized for the treatment of complex neurological disorders, such as AD [23,24].

*Alpinia oxyphylla* (*A. oxyphylla*), the dried ripe fruit of *Alpinia oxyphylla* Miq., has been long been thought to be “multivalent” for commonly treating diarrhea [25], enuresis (i.e., involuntary urination), dementia [26,27,28], inflammation [29], cancer [29], and other disorders [30,31]. Notably, terpene, the main component of *A. oxyphylla*, is regarded as a representative constituent with putative pharmacological activities by phytochemical studies. Recently, several pharmacology studies have indicated that *A. oxyphylla* has definite beneficial effects in ameliorating cognitive disorders and alleviating pathological brain injuries characteristic of neurodegenerative disorders [26,27,32,33,34]. *A. oxyphylla* extracts were shown to have neuroprotective effects against glutamate-induced apoptosis in cortical neurons [35], as well as biological activity towards regulating redox homeostasis, increasing the anti-oxidative properties of enzymes, improving the cholinergic system, and reducing Aβ peptide levels. Moreover, extracts also ameliorated the learning and memory impairment of AD mouse models after being administered by intracerebroventricular injection, which reduced β-amyloid levels significantly [26,36]. However, despite the known therapeutic effects of *A. oxyphylla*, its pharmacological and molecular mechanism of action have not been fully elucidated. Nonetheless, these aforementioned studies have given us a foundation to further study the molecular mechanisms of *A. oxyphylla* using systems pharmacology methodologies.

It is worth mentioning that our previous experiments have verified the neuroprotective effects of *A. oxyphylla* by extending the nonparalytic rate and reducing the mean paralysis rate on amyloid beta (1-42) transgenic *Caenorhabditis elegans* (*C. elegans*) (data not shown). In this paper, pharmacokinetic evaluation, target prediction, network, and pathway analysis using multiple available public databases and bioinformatics resources, have revealed potential pharmacological mechanisms and beneficial effects of *A. oxyphylla* on AD.

## 2. Results

### 2.1. Screening of Candidate Compounds and Predicting Putative Target Proteins

**Figure 1:**
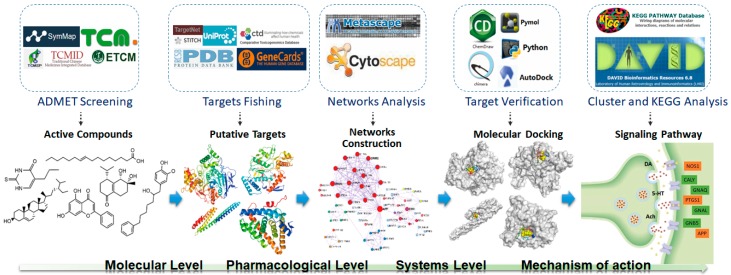
Network pharmacology for deciphering pharmacological mechanisms of *A. oxyphylla* acting on Alzheimer’s disease.

**Figure 2:**
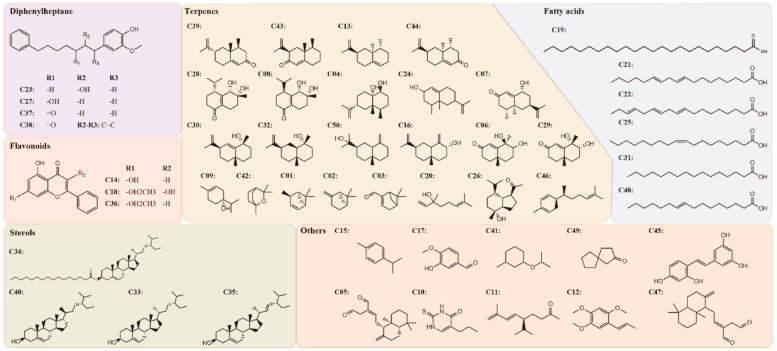
The 2-dimensional (2D) molecular structures and the classification of 50 candidate compounds in *A. oxyphylla*. There are 6 classifications including diphenylheptane (4), flavonoids (3), sterols (4), terpenes (23), fatty acids (6) and others (10).

The potential pharmacological mechanisms and the details behind each step in defining the role of *A. oxyphylla* on AD using system pharmacology are shown in Figure 1. Fifty compounds were selected as candidates by screening and excluding duplicates from the native compounds of *A. oxyphylla* based on the Symmap, TCMSP, TCM-MESH, and TCMID database analyses. All 50 compounds were divided into 6 categories: 23 terpenes (e.g., oxyphyllol A, oxyphyllol B, zingiberol, among others), 6 fatty acids (e.g., lignoceric acid, oleic acid), 4 diphenylpeptanes (e.g., neonootkatol, oxyphyllacinol), 4 sterols (e.g., sitoterpene, sitoterpene palmitate), 3 flavonoids (chrysin, izalpinin, and tectochrysin), and 10 others (e.g., solanone, isovanilin, azaron, among others), as shown in Figure 2. Furthermore, 12 compounds presented a good score in the ADMET criterion, and 18 compounds presented moderate levels after evaluating them in the ETCM database (Supplementary Materials Table S1).

**Figure 3:**
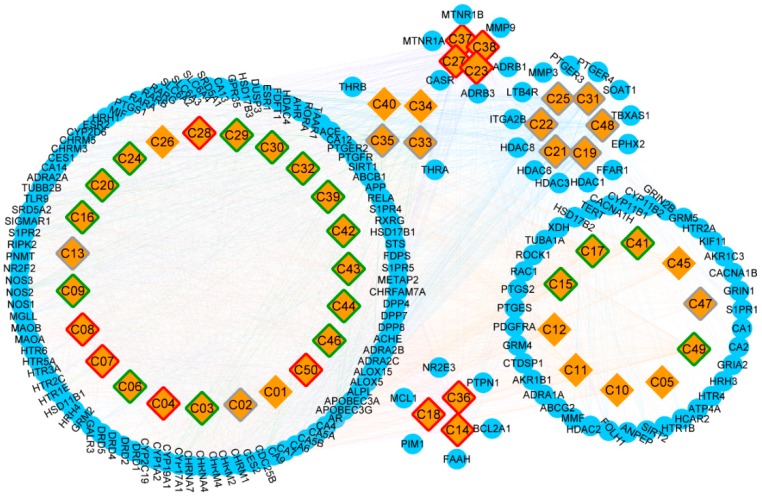
The construction of Compound-Target network, and the classification of putative target proteins of *A. oxyphylla.* The candidate compounds (diamond mesh node with yellow) are divided into groups by structural category, the pharmacokinetic properties of which are divided into good (red border), moderate (green border), week (gray border), and N/A (white border). Similarly, the putative target proteins (the circular mesh node with blue) are grouped and surrounded with the corresponding compounds.

A total of 31150 target proteins were fished along with the 50 candidate compounds using the Lipinski Rule of Five in QSAR and overlapping them with STITCH results. A higher docking probability and combined score of putative target proteins indicated a closely integration with the compounds. Among these target proteins, 164 targets were screened as putative target proteins, 99 of which were putative target proteins of terpenes (Table S2). Furthermore, the CT network was constructed and illustrated based on the various categories of compounds and putative proteins aligned (Figure 3). Compound nodes with red, green, gray, and white borders represented good, moderate, weak, and N/A level of ADMET evaluation respectively. Among these, five terpenes (oxyphyllol B/C, oxyphyllenodiol A/B, and zingiberol), four diphenylpeptanes (neonootkatol, oxyphyllacinol, and yakuchinone A/B), and three flavonoids (chrysin, izalpinin, and tectochrysin) reflected good pharmacokinetic properties by the ADMET criterion. Moreover, our Venn diagram clearly showed the overlapping of terpene proteins, which represent more than half of the protein targets, than any of the other five groups (Figure S1). Thus, we speculated that terpenes may be the predominant and unique primary bioactive substances mediating the neuroprotective effects of *A. oxyphylla.*

### 2.2. Exploration of A. oxyphylla Molecular Mechanism of Action

The 99 putative target proteins of terpenes were selected based on their integration score and GO and KEGG pathway enrichment analyses were initiated. After filtering by a parameter *p*-value cutoff of ≤ 0.01, 579 GO terms and 28 KEGG pathway terms for terpenes were identified, as shown in Tables S3 and S4 (Figure S2D). A total of 579 GO terms were included: 459 for biological processes, 76 for molecular function, and 44 for cellular components (Table S3). In order to show the results of the GO enrichment in an intuitive and explicit way, a bubble diagram was utilized. As shown in Figure S2A–C, *p*-values are given the highest priority in descending order. More importantly, the muscarinic acetylcholine receptor family M1-M5 (CHRM1, 2, 3, 4 and CHRM5), the neuronal acetylcholine receptor subunit alpha-4 and 7 (CHRNA4 and CHRNA7), the 5-hydroxytryptamine receptor 3A and 5A (HTR3A and HTR5A), the sodium-dependent serotonin transporter (SLC6A4), and the dopamine D1/D2 receptor (DRD1 and DRD2) were the most frequently occurring protein targets, mainly enriched in nervous (has04725, 04726, and 04728) and sensory systems (has04742), signaling molecules and interaction (hsa04080), signal transduction (hsa04020, 04022, 04024, 04066, and 04371), as well as substance dependence related signaling pathways, especially in neurotransmission (GO: 0030594, 0099536, and 0099537), neurons and synapse formation (GO: 0030425, 0045211, 0097447, 0097060, and 0099699), serotonin receptor (GO: 0099589), and G protein-coupled receptor (GPCR) signaling pathways (GO: 0004993, 0007187, 0007188, 0008227, and 0099528). Thus, we further identify that terpenes played a key regulating role in nervous system diseases, such as AD.

Furthermore, gene entries related to AD were collected from the CTD and GeneCards databases. As a result, 22150 and 6634 protein coding gene entries were collected from each database, respectively. A total of 251 gene entries with prioritized inference scores and relevance scores were screened and integrated from two databases, which served as the key putative target proteins of AD (Tables S5 and S6). Among the putative target proteins of *A. oxyphylla*, 21 target proteins associated with AD were found, including the amyloid-beta precursor protein (APP), the ATP-dependent translocase ABCB1 (ABCB1) protein, the neuronal acetylcholine receptor subunit alpha-7 (CHRNA7), the angiotensin-converting enzyme (ACE), the glutamate receptor ionotropic N-methyl-D-aspartate receptor (NMDA) 2B (GRIN2B), the cytochrome P450 1A2 (CYP1A2), acetylcholinesterase (ACHE), the 5-hydroxytryptamine receptor 2A (HTR2A), the sodium-dependent dopamine transporter (SLC6A3 and SLC6A4), nitric oxide synthase (brain: NOS1, inducible: NOS2, and endothelial: NOS3), neprilysin (MME), stromelysin-1 (MMP3), the matrix metalloproteinase-9 (MMP9), the NAD-dependent protein deacetylase sirtuin-1 (SIRT1), prostaglandin G/H synthase 1, 2 (PTGS1 and PTGS2), transcription factor p65 (RELA), and glutamate receptor ionotropic, NMDA 1 (GRIN1), as listed in Table S7. The overlap proteins from terpenes and the clinically tested AD-associated target proteins reflect the potential functions of terpenes in treating AD.

### 2.3. Construction and Analysis of the Integrated Network Model

**Figure 4:**
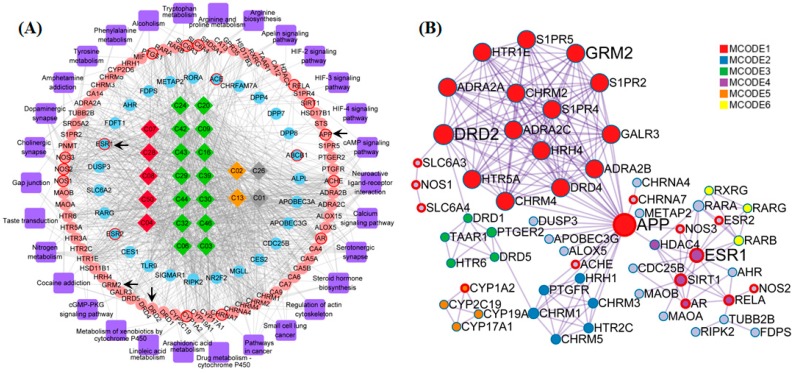
The constructions and analysis of compound-target-pathway (CTP) network and the Protein-Protein Interaction (PPI) network of terpenes. (**A**) The candidate compounds with different pathological properties (good: Red diamond, moderate: Green diamond, week: Yellow diamond and N/A: Gray diamond), the ellipse nodes respect the KEGG enrichment terms with *p* < 0.01 (pink ellipse) and nodes not enriched in KEGG with *p* < 0.01 (blue ellipse), and AD-associated proteins (ellipse with red border) and the pathways (purple round rectangle) of Terpenes are constructed. (**B**) The protein-protein-interaction (PPI) network of Terpenes are illustrated with color by clusters and size by degree. Among these nodes, APP, ESR1, DRD2 and GRM2 are served as core targets with red border.

The compound-target-pathway-disease (CTPD) network of *A. oxyphylla* contained 234 nodes and 2594 edges, with the AD-related putative target proteins marked with a red border, and the other putative target proteins marked in blue, as shown in Figure S3. In order to further inquire into the underlying molecular mechanisms mediating the neuroprotective mechanisms of *A. oxyphylla*, the compound-target-pathway (CTP) and protein–protein interaction (PPI) network of terpene main ingredients were carried out in this research. Specifically, the CTP network model of terpenes contained 152 nodes and 995 edges, with 72 differentially expressed genes (*p* < 0.01) that were enriched in 28 pathways, based on the GO/KEGG pathway enrichment analysis (Figure 4A). The ellipse nodes with red border represent the 13 AD-associated proteins and 4 key proteins (from 13 hubba proteins, Table S8), which were filtered by topological analysis of 164 putative proteins from the HINT hqb as background network (Figure S4). Importantly, the PPI network is the premise and basis to obtain nodes with more substantial contributions. Overlap nodes were screened by valuable topological indices including degree, betweenness centrality, closeness centrality, and bottleneck nodes of the top 20 nodes, which have significant impact on the performance of the whole network. In the PPI network, the estrogen receptor (ESR1), APP, D2 dopamine receptor (DRD2), and metabotropic glutamate receptor 2 (GRM2) were filtered as bottleneck node proteins consistent with their molecular functions. Clustering and topology approaches were utilized to identify individual variations and similarities among various protein targets (Figure 4B).

Furthermore, it is of great significance for compounds with better water solubility, blood–brain barrier permeability, and pharmacokinetic properties in thinking of drug design and development. For better understanding of the mechanism of action and structure-activity relationship of terpenes and its putative targets, terpene compounds with good pharmacokinetic properties by the ADMET screening criteria and its common targets (i.e., ACHE, AR, HTR1E, NOS2, SRD5A2, CHRM4, and CYP17A1) were selected. Among these target proteins, ACHE and NOS2 were found to be the overlapping nodes with AD- associated proteins, which is valuable for future studies.

### 2.4. Molecular Docking

In order to further verify the functional effects of terpenes in AD, the interaction between the active compounds of terpenes with good pharmacokinetic properties and key putative proteins were tested and verified randomly. With the application of AutoDockTools-1.5.6, Discovery Studio 4.5 Client, and Pymol software, the interactions among the potential active compounds (i.e., oxyphyllol B, oxyphyllenodiol A, eucalyptol, and zingiberol), the AD-associated proteins (i.e., ACHE and NOS2), as well as the bottleneck node proteins (i.e., APP and ESR1) were elucidated. Notably, the lower of binding energy meant the binding between the compounds and the targets were stronger. The Van der Waals forces, hydrogen bonding, and aromatic stacking (Pi-Sigma, Pi-alkyl, and alkyl interactions) were found to be involved between the active site residues and the potential compounds.

**Figure 5:**
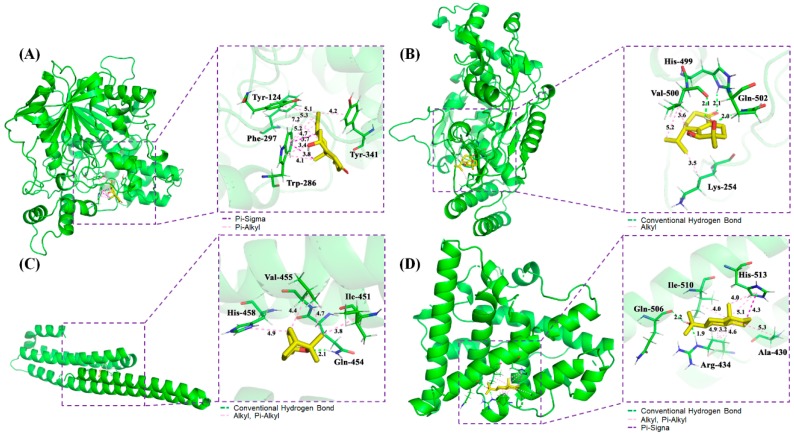
Schematic 3D representation that the molecular docking model, active sites and binding distances, and the ray tracing of compound (**A**) oxyphyllol B (C07) in the protein ACHE (PDB ID: 6o4w), (**B**) oxyphyllenodiol A (C08) with NOS2 (4nos), (**C**) eucalyptol (C42) with APP (5buo), and (**D**) zingiberol (C50) with ESR1 (3os8 revised mutation site), respectively.

The 3D mode and 2D dimensional representation of oxyphyllol B (C07) in the active site of ACHE (6o4w) is represented in Figure 5A and Figure S5A. Interestingly, the free binding energy of C07 with ACHE was found to be ‒4.8 kcal/mol. In addition, C07 showed two Pi-Sigma bond interactions with TRP 286, and eight Pi-alkyl bonds with TYR 124, PHE 297, TRP 286, and TYR 341. The interaction types and distance of binding complexes are also depicted in the enlarged drawing in Figure 5A. The following atoms, TYR 72, PHE 295, VAL 294, ARG 296, SER 293, and LEU 76 were served as the pocket atoms held together around the C07 compound by Van der Waals forces. Similarly, the binding interaction of oxyphyllenodiol A (C08) and NOS2 (4nos) is shown in Figure 5B and Figure S5B. The free binding energy of C08 with NOS2 was found to be -4.68 kcal/mol. In particular, C08 showed 3H-bond interactions with HIS 499, GLN 502, and VAL 500, respectively. VAL 500 and LYS 254 also presented alkyl interactions with C08. Additionally, THR498, GLN 310, GLY313, and ASP131 were able to bind with C08 by Van der Waals forces. The interaction types and distance of these binding complexes are also depicted in the Ray tracing diagram in Figure 5B. Moreover, the binding sites, interaction types and distances, as well as the active atoms of APP (5buo) with eucalyptol (C42) were analyzed and shown in Figure 5C and Figure S5C. The free binding energy of eucalyptol (C42) with APP (5buo) was found to be ‒3.9 kcal/mol. C42 showed 1H-bond interactions with GLN 454 and three alkyl, as well as Pi-alkyl interactions with ILE 451, VAL 455, and HIS 458, respectively. Meanwhile, C42 presented four Van der Waals forces among MET 383, HIS 436, THR 433, and GLU 387. The 2D/3D mode and the interaction types and distance of zingiberol (C50) in the active site of ESR1 (3os8 revised mutation site) is represented in Figure 5D and Figure S5D. The free binding energy of C07 with ACHE was found to be ‒5.28 kcal/mol. C50 showed 2H-bond interactions with ARG 434 and GLN 506, as well as one Pi-Sigma bond with HIS 513. Moreover, C50 also had seven Alkyl and Pi-Alkyl interactions with HIS 513, ALA 430, ILE 510, and ARG 434, respectively. There were two Van der Waals forces of THR 431 and LEU 509 with C50.

## 3. Discussion

TCM has been shown to be an effective treatment for the relieve of complicated diseases in a multi-target/multi-component manner, which makes it unique among all traditional medicines [37]. Importantly, TCM has been used in the treatment of neurological diseases for over thousands of years. The *A. oxyphylla* plant belongs to the Zingiber of Zingiberaceae genus, which is rich in terpenes, flavonoids, and diphenylpeptanes, among other compounds, which have been shown to have clinical activity in patients with diarrhea and enuresis [38,39,40,41]. Recently, a growing number of clinical trials and studies have shown that *A. oxyphylla* has a definite positive effect in ameliorating cognitive impairment and improving the characteristic pathological brain injuries in AD [26,37], although the underlying mechanism of action remains elusive. In addition, our previous results have shown that *A. oxyphylla* extracts have significant neuroprotective effects on reducing the paralysis ratio in AD transgenic *C. elegans*. (data not shown). This prompted us to apply an integrative and computational system pharmacology approach, as well as classical molecular dynamics and molecular docking models to explore the effective substances, putative targets, and potential pharmacological mechanisms of *A. oxyphylla* with its bioactive compounds for treating AD.

**Figure 6:**
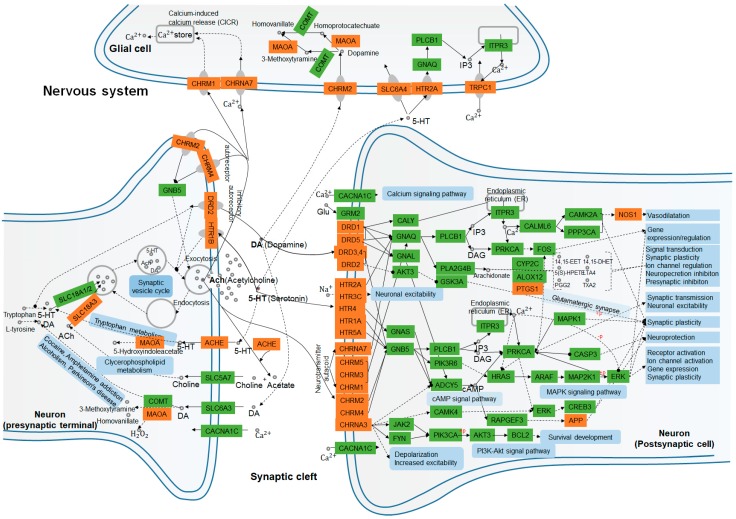
Nervous system interaction effects of the KEGG enrichment pathway terms and putative target proteins identified in *A. oxyphylla.* and terpenes. Pathways and biological functions (blue rectangles) form the compressed nerve synapses signal transmission network. Orange and green rectangles represent the putative target proteins which are identified in this study, and the relative functional proteins in network, respectively. Arrows indicate activation; round-arrows indicate inhibition.

By incorporating SymMap, TCMSP, TCMID, TCM-ID, and ETCM databases, 50 candidate compounds were screened using the ADMET criteria, and 164 putative targets of *A. oxyphylla* (including 99 targets of terpenes) were selected using the QSAR-TargetNet screening criteria. The classification of compounds and the analysis of the compound-target network indicated that terpenes, flavonoids, and diphenylpeptanes with good pharmacokinetic properties served as the main compounds in *A. oxyphylla,* which is consistent with the existing experimental data. Subsequently, 579 GO and 28 KEGG pathway terms, along with 6 protein-associated diseases of *A. oxyphylla* were identified and classified. The functions of these putative target proteins included, G protein-coupled amines, neurotransmitter and serotonin receptor function proteins, dendrite and synaptic membrane components, and proteins involved in GPCR signaling pathways. The GO/KEGG enrichment pathway therefore underscores the potential relevance of the putative proteins of *A. oxyphylla* and AD. We then examined the link between the putative proteins and AD-associated proteins from CTD and GeneCards by CTPD network construction of *A. oxyphylla*, as well as CTP and PPI network construction of terpenes. Among the putative target proteins of *A. oxyphylla,* 21 target proteins associated with AD and 8 putative target proteins (HDAC1, ESR1, EGFR, RELA, ESR2, AR, RAC1, TP53) had the characteristics of bottleneck nodes by topology analysis. Furthermore, the putative target proteins of terpenes, which occupied a great part of the putative targets of *A. oxyphylla*, were annotated with KEGG pathways, and constructed as a protein–protein interaction network for the protein function prediction by clustering algorithm. Based on the manual annotation map, terpenes demonstrated a function on regulating the synthesis and release of neurotransmitters, as well as signal transmission, dendritic growth, and spine formation, including synaptic plasticity in the nervous system (Figure 6).

The left panel of Figure 6 shows that SLC18A1, 2, and 3 may have a role in synaptic vesicle cycling, acetylcholinesterase (ACHE) [42], and amine oxidase [flavin-containing] A (MAOA) [43,44] signaling, which have been shown to be involved in tryptophan metabolism, clycerophospholipid metabolism, and also related to cocaine and amphetamine addiction, as well as alcoholism, and Parkinson’s disease in the presynaptic nerve terminal. Neurotransmitters, such as dopamine (DA, hsa04728), serotonin (5-HT, hsa04726), and acetylcholine (ACh, has04725) work on muscarinic acetylcholine receptor M2 (CHRM2) [45] and MAOA for DA metabolism in glial cells, as well as on CHRNA7 [46] and CHRM1 [47] for calcium (Ca^2+^) storage by Ca^2+^-induced Ca^2+^ release (CICR) [48], and on HTR2A-SLC6A4-IP3-TRPC1 [49,50] pathway for Ca^2+^ transport, respectively. On the postsynaptic cell membrane, DA, is the prototypical slow neurotransmitter of the mammalian brain, which interacts with D1-like receptors DRD1 and DRD5 [51,52], both positively coupled to adenylyl cyclase (AC) and cAMP production, which are activated and regulated downstream of PTGS1 and NOS1 expression. While the activation of D2-like receptors DRD2, DRD3, and DRD4 have exactly the reverse effect on regulating the production of AC and cAMP in dopaminergic synapse pathway (hsa04728) [51] activity. More evidence has suggested that DA influences neuronal activity, synaptic plasticity, and behavior by diverse cAMP- and Ca^2+^-dependent and independent mechanisms (has 04020). Similarly, ACh binds and activates muscarinic acetylcholine receptors CHRM1, 2, 3, 4, and 5 [53], and directly alters cellular homeostasis of phospholipase C, inositol trisphosphate, cAMP, free Ca^2+^, and the activation of neuronal ACh receptors CHRNA4 and 7 [54]. It also regulates the rapid influx of sodium (Na^+^) and Ca^2+^, leading to the subsequent cellular depolarization by cAMP (hsa04024), MAPK (hsa04010), and phosphoinositide 3-kinase (PI3K)/AKT (hsa04151) signaling pathways. For monoamine neurotransmitters, serotonin (5-hydroxytryptamine, 5-HT) binds to ionotropic 5-HT3 receptors, GPCR 5-HT receptors, 5-HT1 (Gi/Go -coupled), 5-HT2 (Gq-coupled), 5-HT4/6/7 (Gs-coupled), and 5-HT5 receptors, which can be directly or indirectly involved in the regulation of synaptic transmission, neuronal excitability, synaptic plasticity, and neuroprotection [55,56]. Therefore, we hypothesize that *A. oxyphylla* or terpenoids are natural compounds that may play an important role in mediating anti-AD effects by functional annotation and enrichment analysis of neurotransmitter receptors.

Based on our topology analysis, hubba nodes screened, and protein–protein interaction network constructions of terpenes, two bottleneck node proteins, APP [57] and ESR1 [58], and two AD-associated targets, ACHE [59] and NOS2 [60], were chosen as the basis for the compound-ligand interaction analysis by AutoDock for preliminary studies aimed at investigating the anti-AD effects of terpenes. The good molecular docking scores and results reflected that terpenes possess suitable anti-AD activity. Other putative protein targets are also highly matched with several representative compounds in *A. oxyphylla*, however the specific actions and properties need to be further verified and probed in future studies.

## 4. Materials and Methods

### 4.1. Compound Database Building

In the present study, the chemical compound data of *A. oxyphylla* were collected from SymMap (http://symmap/org/) [61], which is an integrative database covering information from the Traditional Chinese Medicine System Pharmacology (TCMSP) database and analysis platform, http://ibts.hkbu.edu.hk/LSP/tcmsp.php) [62], the Traditional Chinese Medicine Integrated Database (TCMID) [63] the Traditional Chinese Medicine Information Database (TCM-ID) [64], and the Encyclopedia of Traditional Chinese Medicine (ETCM; http://www.nrc.ac.cn:9090/ETCM/) database [65]. The comprehensive drug-likeness grading and pharmacokinetic properties of *A. oxyphylla* were evaluated and filtered for the candidate compounds using the absorptions, distribution, metabolism, excretion, and toxicity (ADMET) criterion as oral bioavailability (OB ≥ 30%), drug-likeness (DL ≥ 0.18) evaluation, and blood–brain barrier (BBB ≥ −0.3), respectively. In addition, the two/three dimensional (2D/3D) structure, canonical smiles, and PubChem ID of the compounds were calibrated using the PubChem (https://pubchem.ncbi.nlm.nih.gov/) database.

### 4.2. AD Associated Proteins and Putative Target Protein Screening

AD-associated protein targets were identified using the comparative toxicogenomics database (CTD; http://ctdbase.org/) [66] and the GeneCards (http://www.genecards.org/) [67] database, with higher rank score, which respected the higher correlation with AD. Compounds were then scored using the Lipinski Rule of Five, and the putative target protein screening, which were fishing and filtered conditionally from the quantitative structure activity relationships-TargetNet (QSAR-TargetNet: http://targetnet.scbdd.com) [68]. The AD-associated proteins and putative target proteins of candidate compounds were verified by their unique UniProtKB ID and target names in the UniProt database (http://www.uniprot.org/) [69,70].

### 4.3. Gene Ontology (GO) and Kyoto Encyclopedia of Genes and Genomes (KEGG) Pathway Enrichment and Network Constructions

Metascape, a web-based portal for comprehensive gene annotation and analysis resources (http://metascape.org/) [71,72], combines a GO [73] and KEGG [74] pathway enrichment analysis search to leverage over 40 independent knowledge bases. The GO/KEGG pathway enrichment terms of the putative proteins with a *p*-value of ≤ 0.01 were regarded as significant and of interest.

In order to clarify the pathogenesis of AD and elucidate the mechanism of action of *A. oxyphylla*, the compound-target interaction (CT), compound-target-pathway (CTP), and compound-target-pathway-disease (CTPD) network were constructed and analyzed using topological parameters and the subnetwork of the bottleneck nodes were filtered and visualized using the Cytoscape 3.6.0 software (Institute for Systems Biology, Seattle, WA, USA; http://www.cytoscape.org/) [75]. Protein–protein interactions (PPI) were set up on Metascape and STRING (https://string-db.org/) [76] databases and also visualized using Cytoscape by cluster analysis.

### 4.4. Molecular Docking

The binding ability, sites, and interactions between compound and target proteins were achieved and analyzed by classical molecular dynamics using AutoDockTools-1.5.6, Pymol 2.3 and Discovery Studio 4.5 Client [77,78]. The 3D chemical structural formulas of candidate compounds were obtained from PubChem and energy minimizing employed to ChemBioDraw 3D. In addition, the crystal structures of putative targets were obtained from the Protein Data Bank (http: //www.pdb.org/) and decorated by removing the ligands and water motifs, revising it and optimizing the mutation sites, and adding hydrogen through the Pymol 2.3 and UCSF Chimera 1.14rc software.

## 5. Conclusions

In summary, *A. oxyphylla* has been shown to exert positive effects in the treatment of AD. In this study, we used network pharmacology and molecular docking approaches to investigate the mechanism of its anti-AD activity. Our findings show that *A. oxyphylla* as TCM, displays a holistic performance through multiple pathways with multi-targets. *A. oxyphylla*, especially terpenes, appear to possess neuroprotective effects on regulating the synthesis, release, and transmission of neurotransmitters, as well as in the formation and plasticity of dendritic spines and synapses in the nervous system, which contributes, as a theoretical basis, to new insights for the development of novel anti-AD drugs.

## Supplementary Materials

The following are available online at https://www.mdpi.com/1422-0067/21/6/2071/s1, Figure S1: Venn diagram illustrated that the six overlapping classifications of 164 putative target proteins which consistent with the compounds in *A. oxyphylla.* Figure S2: Gene Ontology (GO) and Kyoto Encyclopedia of Genes and Genomes (KEGG) pathway enrichment analysis for the top 20 putative target proteins of terpenes. Figure S3: The constructions and analysis of Compound-Target-Pathway-Disease (C-T-P-D) network of *A. oxyphylla.* Figure S4: The network topology analysis of *A. oxyphylla.* Figure S5: The molecular docking results of compounds (oxyphyllol B, oxyphyllenodiol A, eucalyptol and zingiberol) interacted with proteins (ACHE, NOS2, APP and ESR1), respectively. Table S1: Result of 50 candidate compounds of *A. oxyphylla.* Table S2: Result of 164 putative target proteins of *A. oxyphylla.* Table S3: Result of 597 GO terms enrichment of terpenes in *A. oxyphylla.* Table S4: Result of 28 KEGG terms enrichment of terpenes in *A. oxyphylla.* Table S5-1: AD-associated target proteins information collected from Comparative Toxicogenomics Database (CTD). Table S5-2: 164 AD-associated target proteins of Homo sapiens from CTD with an Inference Score of ≥50 were regarded as the more substantially contributing proteins. Table S6-1: AD-associated target proteins information collected from The Human Gene Database (GeneCards). Table S6-2: 118 AD-associated target proteins of Homo sapiens from Genecards with an Inference Score of ≥30 were regarded as the more substantially contributing proteins. Table S7: Result of the KEGG pathway enrichment analysis of *A. oxyphylla.* Table S8: 13 hubba node proteins of *A. oxyphylla*.
