# Supplementary material for: Pharmacological Mechanisms Underlying the Neuroprotective Effects of Alpinia oxyphylla Miq. on Alzheimer’s Disease
Source: Int J Mol Sci. 2020 Mar 18;21(6):2071. doi: 10.3390/ijms21062071 (PMC7139528; doi:10.3390/ijms21062071)
Supplement: Supplementary file 1 [file ijms-21-02071-s001.zip › Supplementary Materials-v6.-revised/Supplementary Figures-revised.docx]

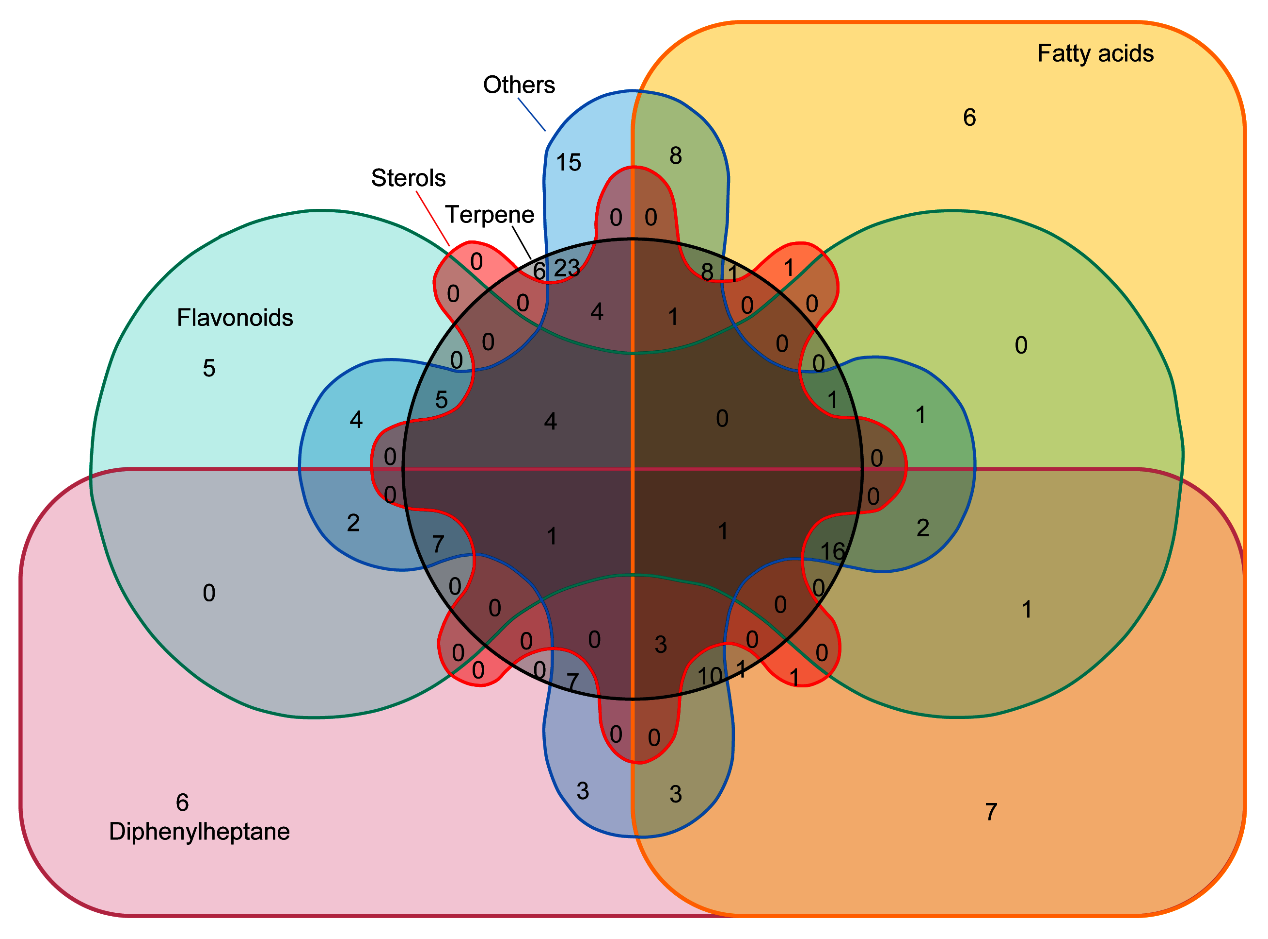


**Figure S1** Venn diagram illustrated that the six overlapping classifications of 164 putative target proteins which consistent with the compounds in *A. oxyphylla.* Candidate compounds are divided into six overlapping classifications, as diphenylheptane in purple, fatty acids in orange, flavonoids in green, sterols in red, others in blue and terpene in dark, respectively.


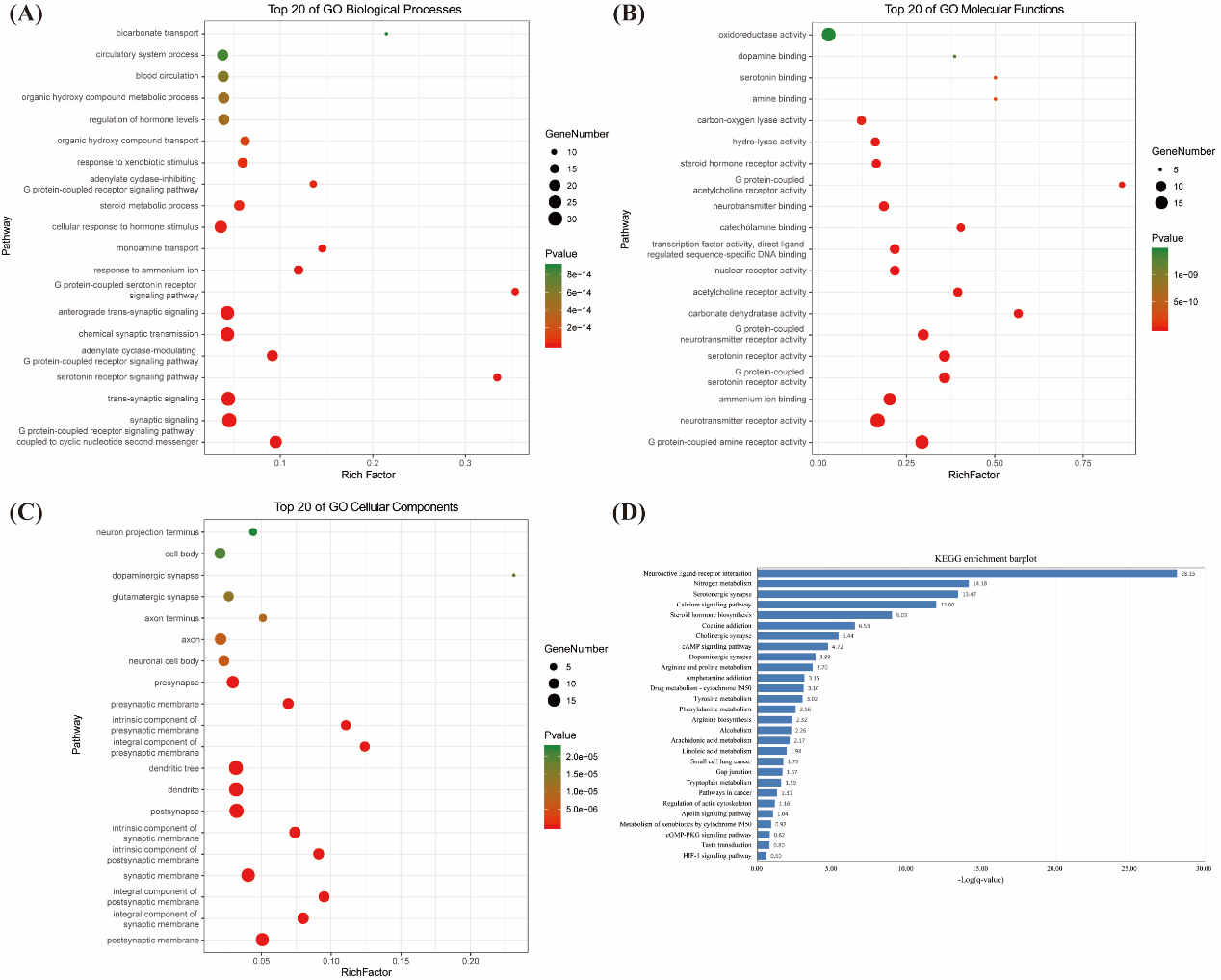


**Figure S2** Gene Ontology (GO) and Kyoto Encyclopedia of Genes and Genomes (KEGG) pathway enrichment analysis for the top 20 putative target proteins of terpenes. The significantly enrichment of (A) Biological Process (BP), (B) Molecular Functions (MF), (C) Cellar Components (CC) categories in GO relative to the target genes with *p*-value ≤ 0.01. The KEGG enrichment terms with *p*-value ≤ 0.01 are showed in Figure S2 D.


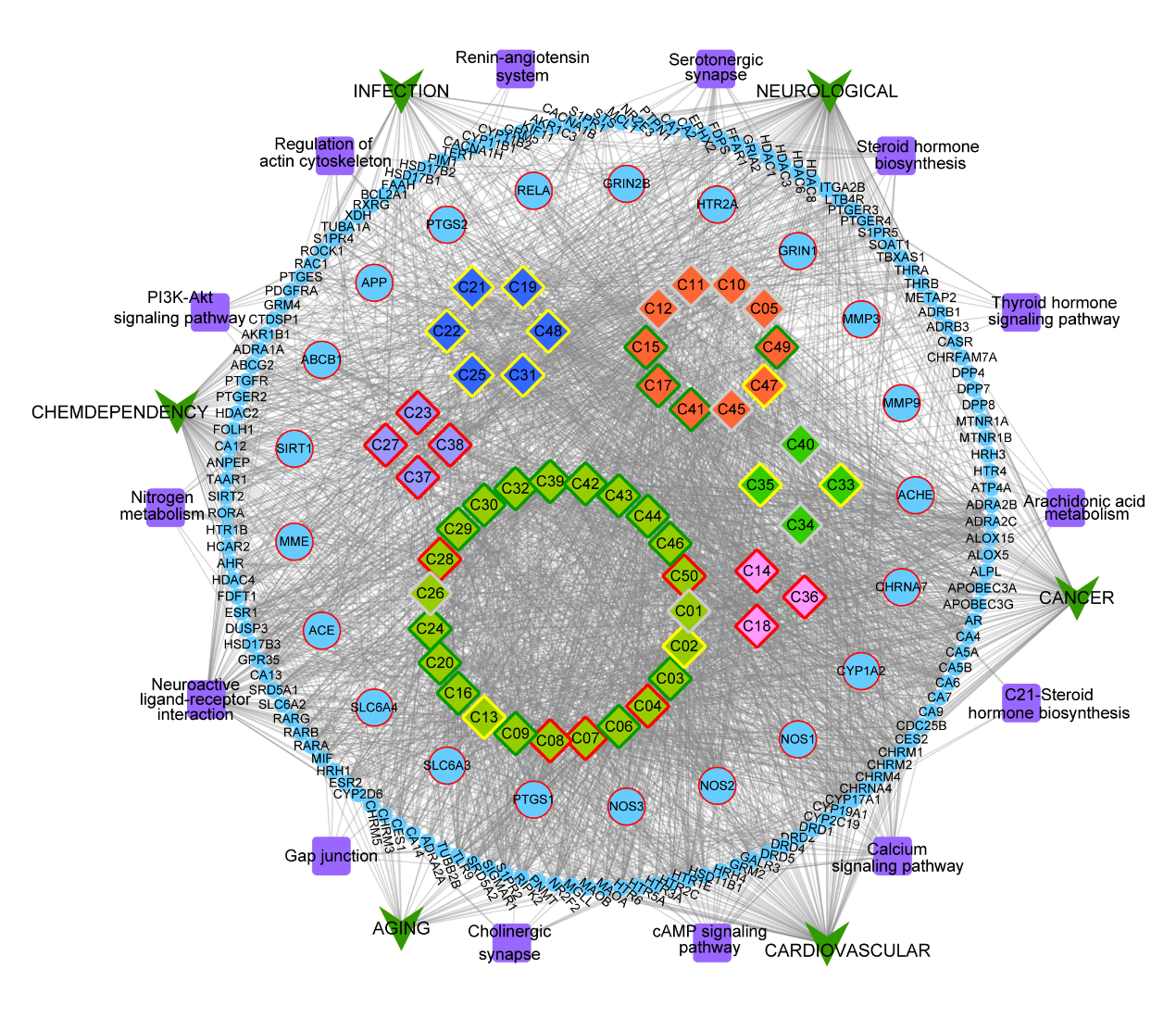


**Figure S3** The constructions and analysis of Compound-Target-Pathway-Disease (C-T-P-D) network of *A. oxyphylla*. Candidate compounds (diamond mesh node) with various categories (diphenylheptane: blue, flavonoids: pink, Sterols: green, terpenes: chartreuse, fatty acids: navy, and others: orange) and different pathological properties (good: red border, moderate: green border, week: gray border and N/A: white border), putative targets (blue ellipse) and AD-associated proteins (blue ellipse with red border), pathways (purple round rectangle) and diseases (green V) formed the C-T-P-D network of *A. oxyphylla.*


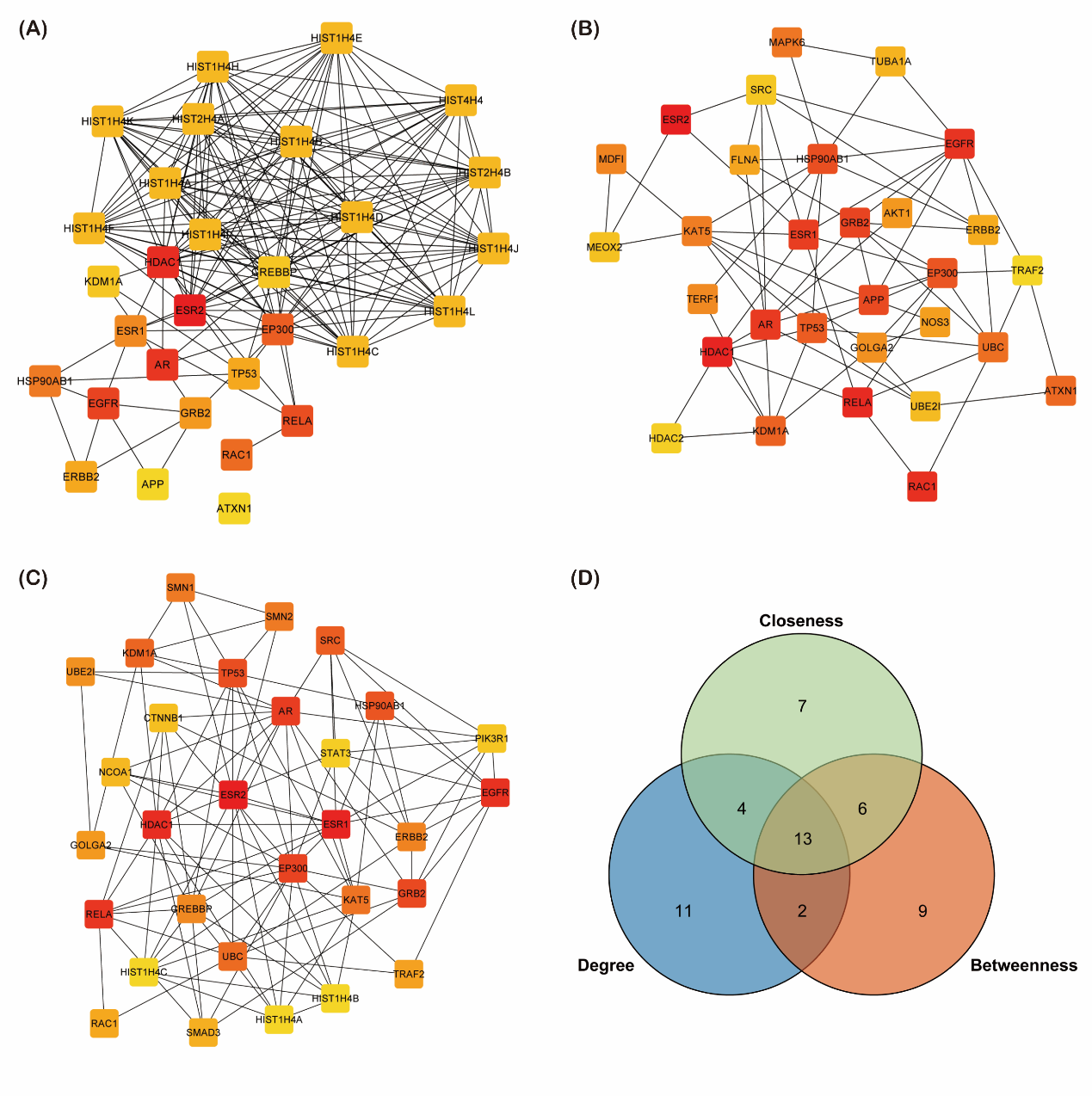


**Figure S4** The network topology analysis of *A. oxyphylla.* The illustrations of the network topology analysis including the top 30 nodes from degree (A), betweenness (B) and closeness (C) subnetworks of *A. oxyphylla*, and the overlaps between degree, closeness and betweenness relative proteins by Veen diagram (D).


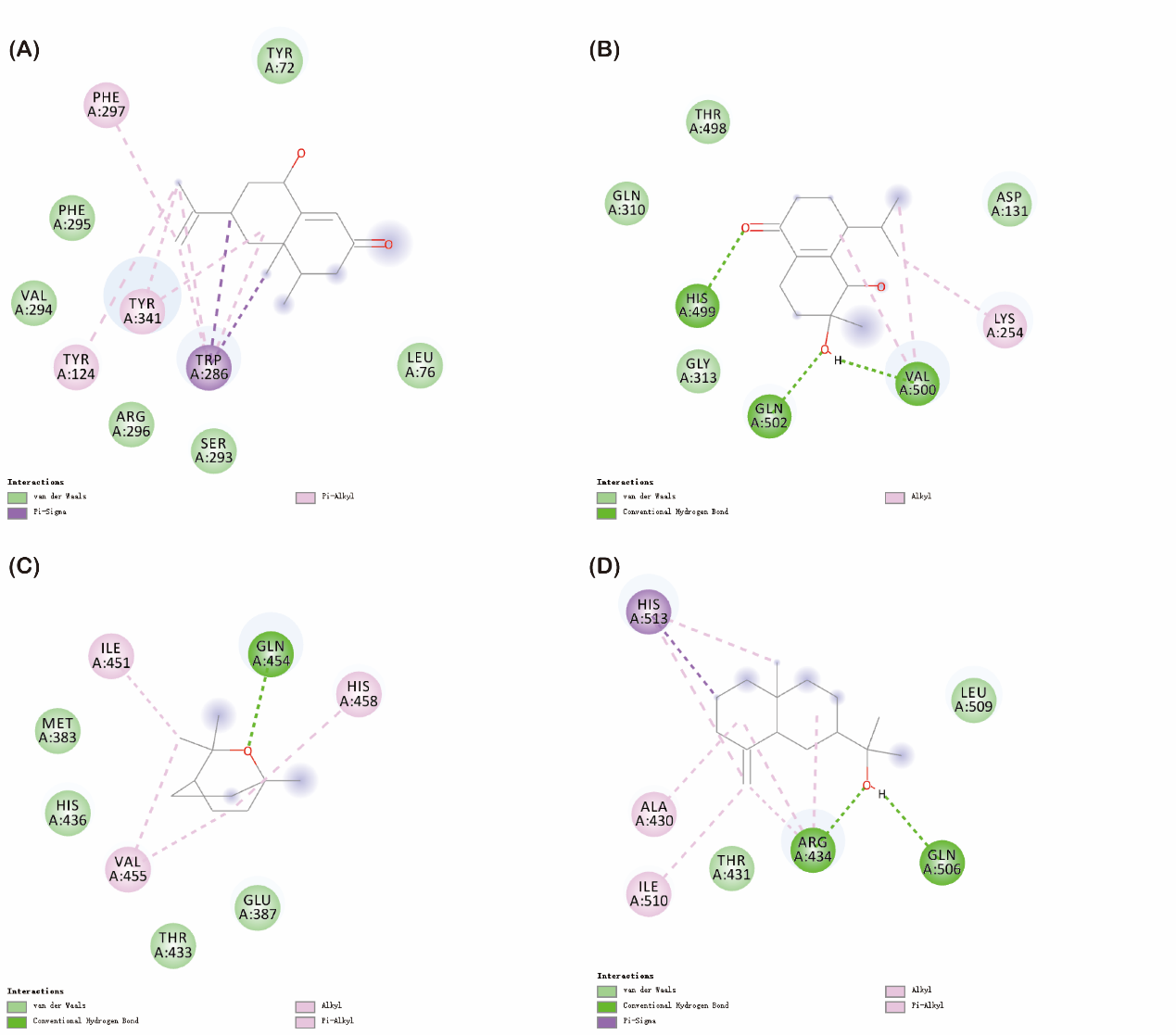


**Figure S5** The molecular docking results of compounds (oxyphyllol B, oxyphyllenodiol A, eucalyptol and zingiberol) interacted with proteins (ACHE, NOS2, APP and ESR1), respectively. Schematic 2D representation that the molecular docking model, active sites and the types of interaction in compounds (panel A: oxyphyllol B (C07), panel B: oxyphyllenodiol A (C08), panel C: eucalyptol (C42) and panel D: zingiberol (C50)) corresponding proteins (ACHE, NOS2, APP and ESR1), respectively.
