# Supplementary material for: Pharmacological Mechanisms Underlying the Neuroprotective Effects of Alpinia oxyphylla Miq. on Alzheimer’s Disease
Source: Int J Mol Sci. 2020 Mar 18;21(6):2071. doi: 10.3390/ijms21062071 (PMC7139528; doi:10.3390/ijms21062071)
Supplement: Supplementary file 1 [file ijms-21-02071-s001.zip › Supplementary Materials-v6.-revised/~WRL2402.tmp]

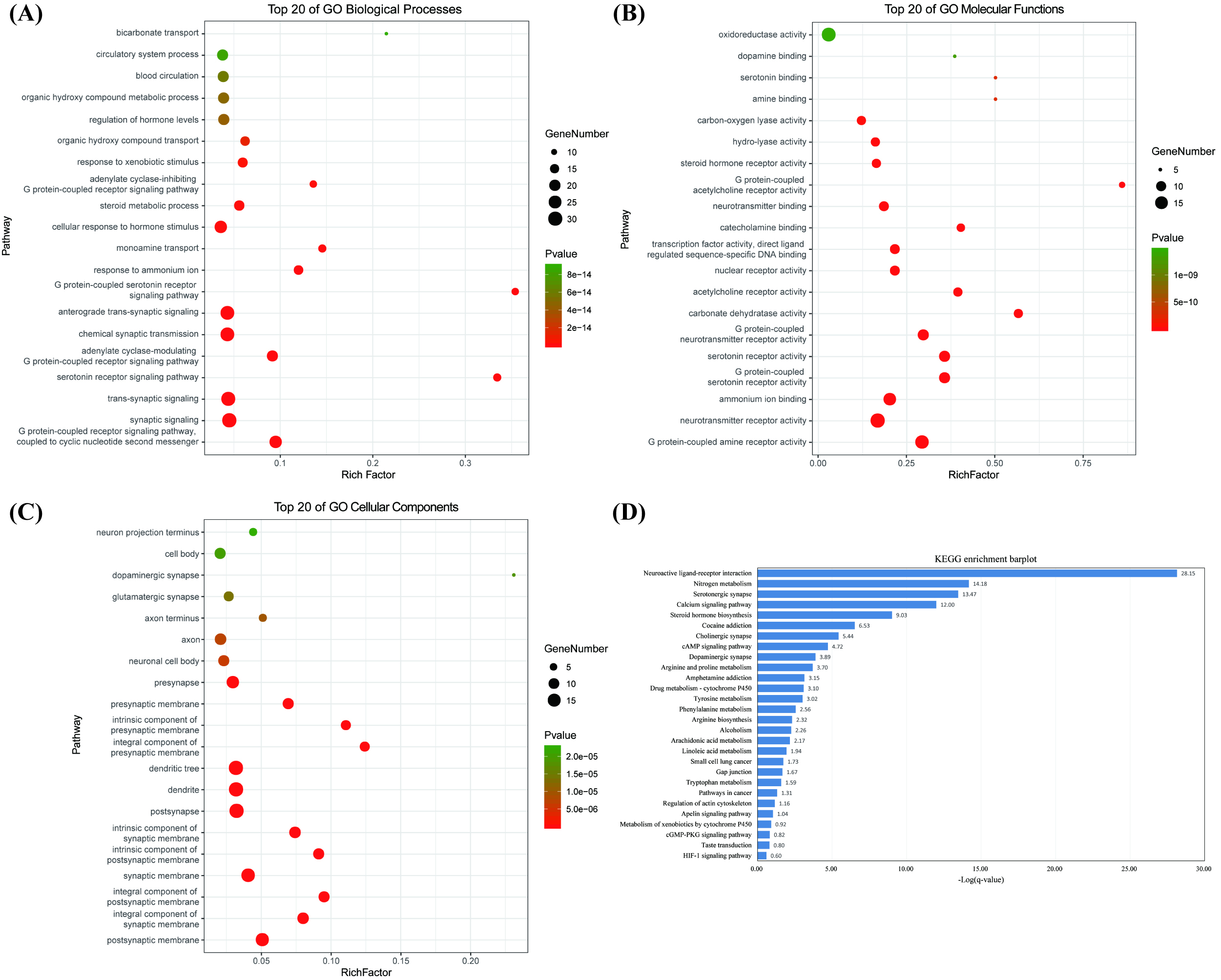


**Figure S1** Gene Ontology (GO) and Kyoto Encyclopedia of Genes and Genomes (KEGG) pathway enrichment analysis for the top 20 putative target proteins of terpenes. The significantly enrichment of (A) Biological Process (BP), (B) Molecular Functions (MF), (C) Cellar Components (CC) categories in GO relative to the target genes with *p*-value ≤ 0.01. The KEGG enrichment terms with *p*-value ≤ 0.01 are showed in Figure S1 D.

**Figure S2** The illustrations of the network topology analysis including the top 30 nodes from degree (A), betweenness (B) and closeness (C) subnetworks of *A. oxyphylla*, and the overlaps between degree, closeness and betweenness relative proteins by Veen diagram (D).


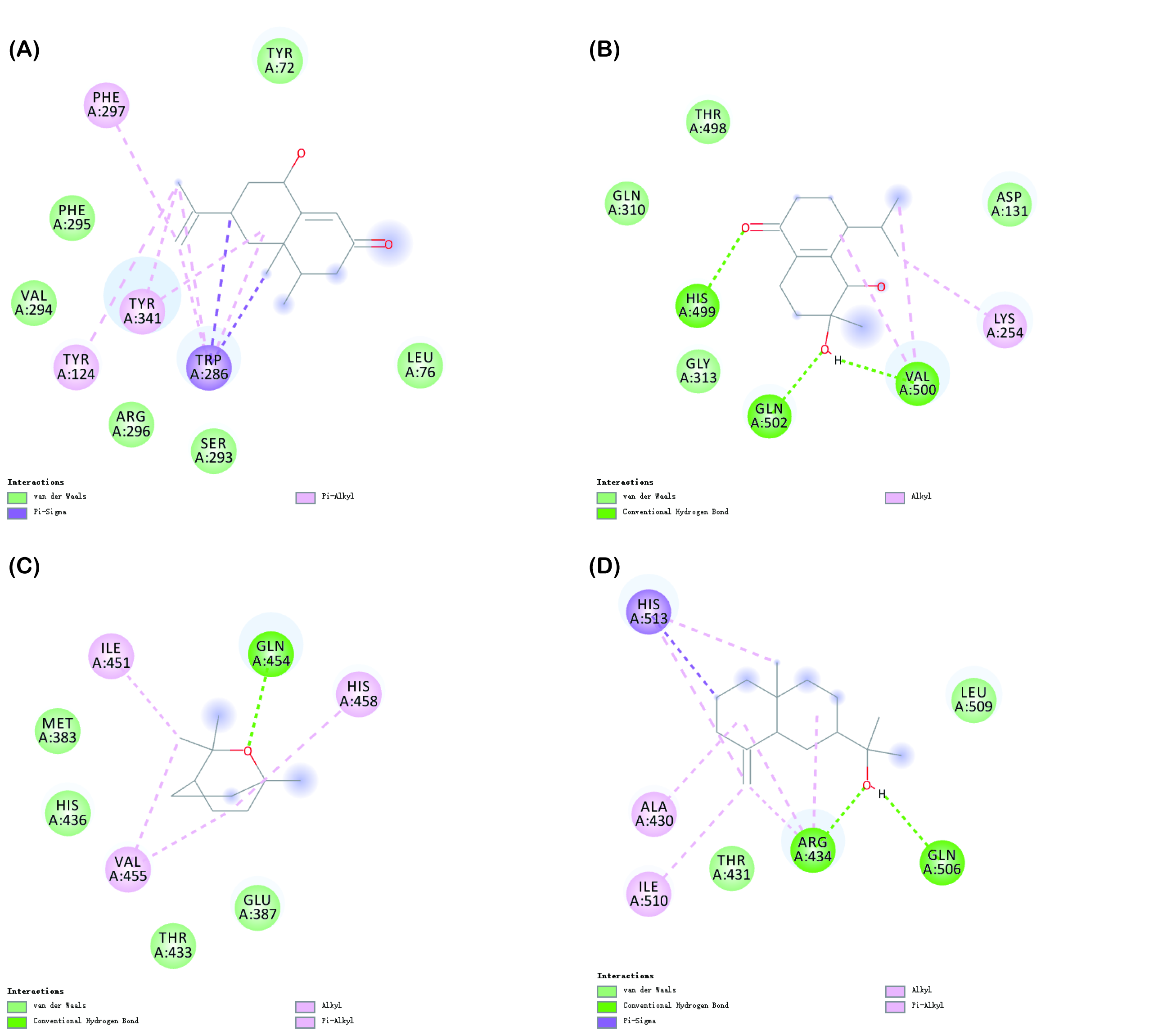


**Figure S3** Schematic 2D representation that the molecular docking model, active sites and the types of interaction in compounds (panel A: oxyphyllol B (C07), panel B: oxyphyllenodiol A (C08), panel C: eucalyptol (C42) and panel D: zingiberol (C50)) corresponding proteins (ACHE, NOS2, APP and ESR1), respectively.
