# Supplementary figures and images for: Pharmacological Mechanisms Underlying the Neuroprotective Effects of Alpinia oxyphylla Miq. on Alzheimer’s Disease
Source: Int J Mol Sci. 2020 Mar 18;21(6):2071. doi: 10.3390/ijms21062071 (PMC7139528; doi:10.3390/ijms21062071)

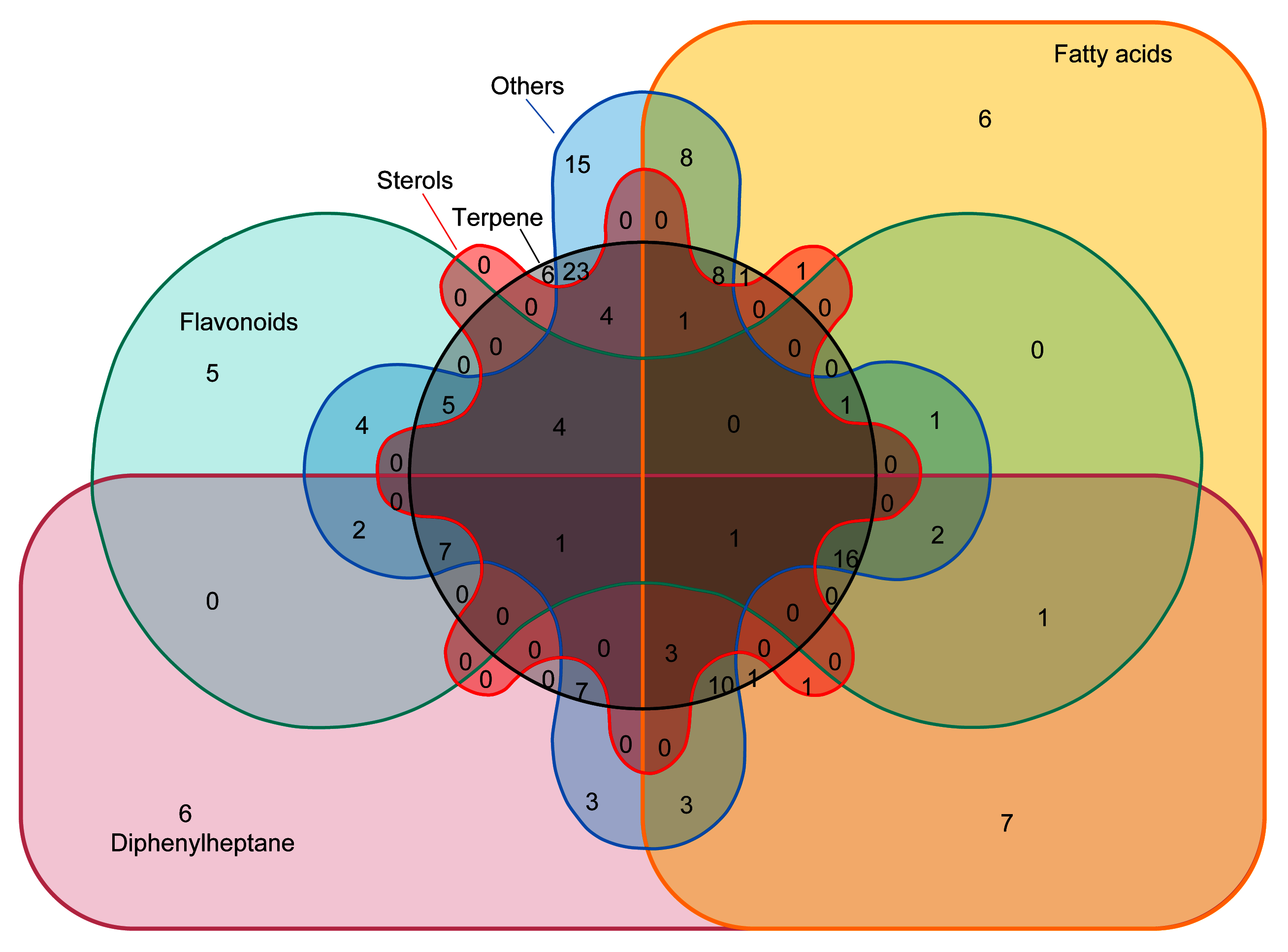

Supplement: Supplementary file 1 [file ijms-21-02071-s001.zip › Supplementary Materials-v6.-revised/Figure S1.tif]

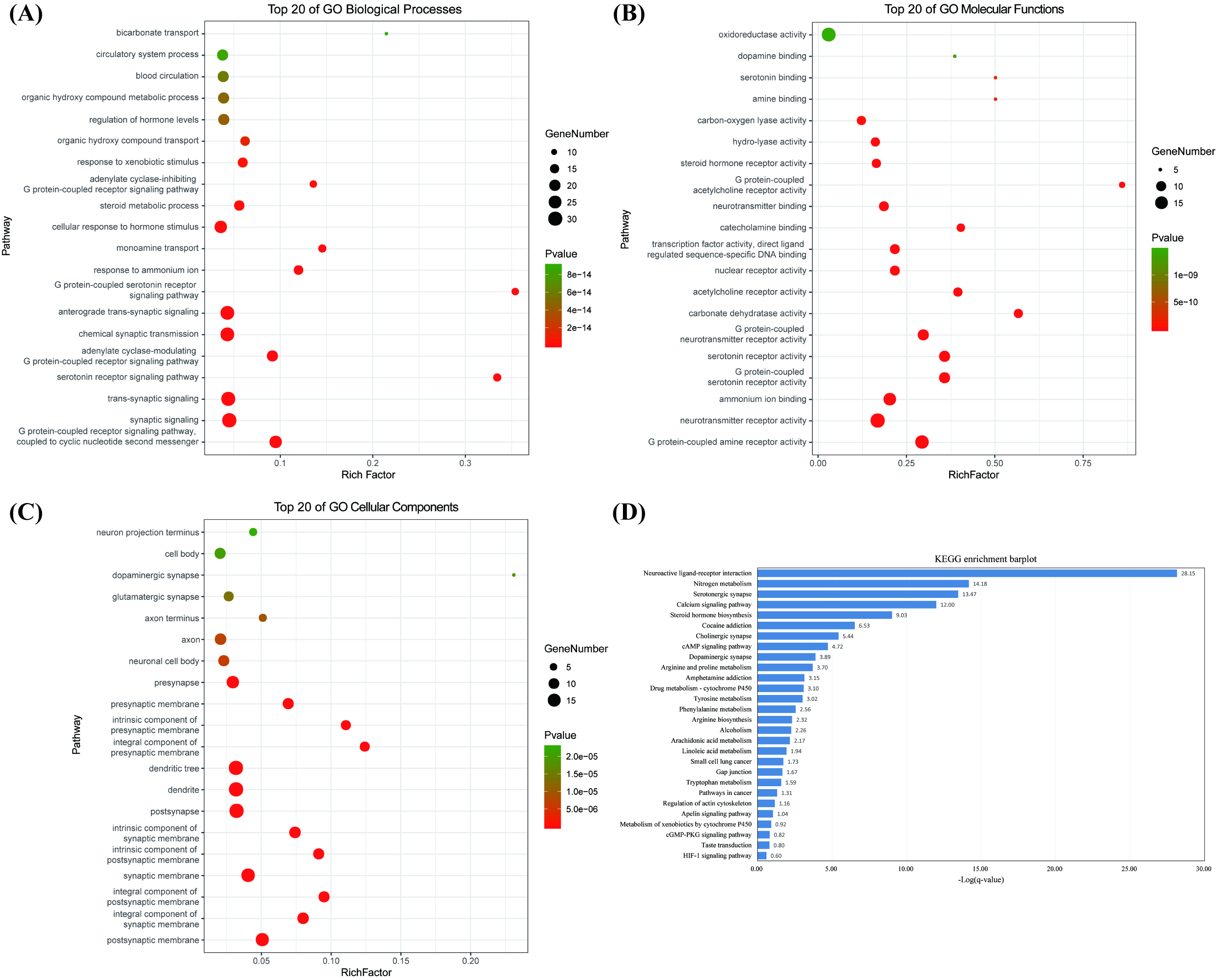

Supplement: Supplementary file 1 [file ijms-21-02071-s001.zip › Supplementary Materials-v6.-revised/Figure S2.tif]

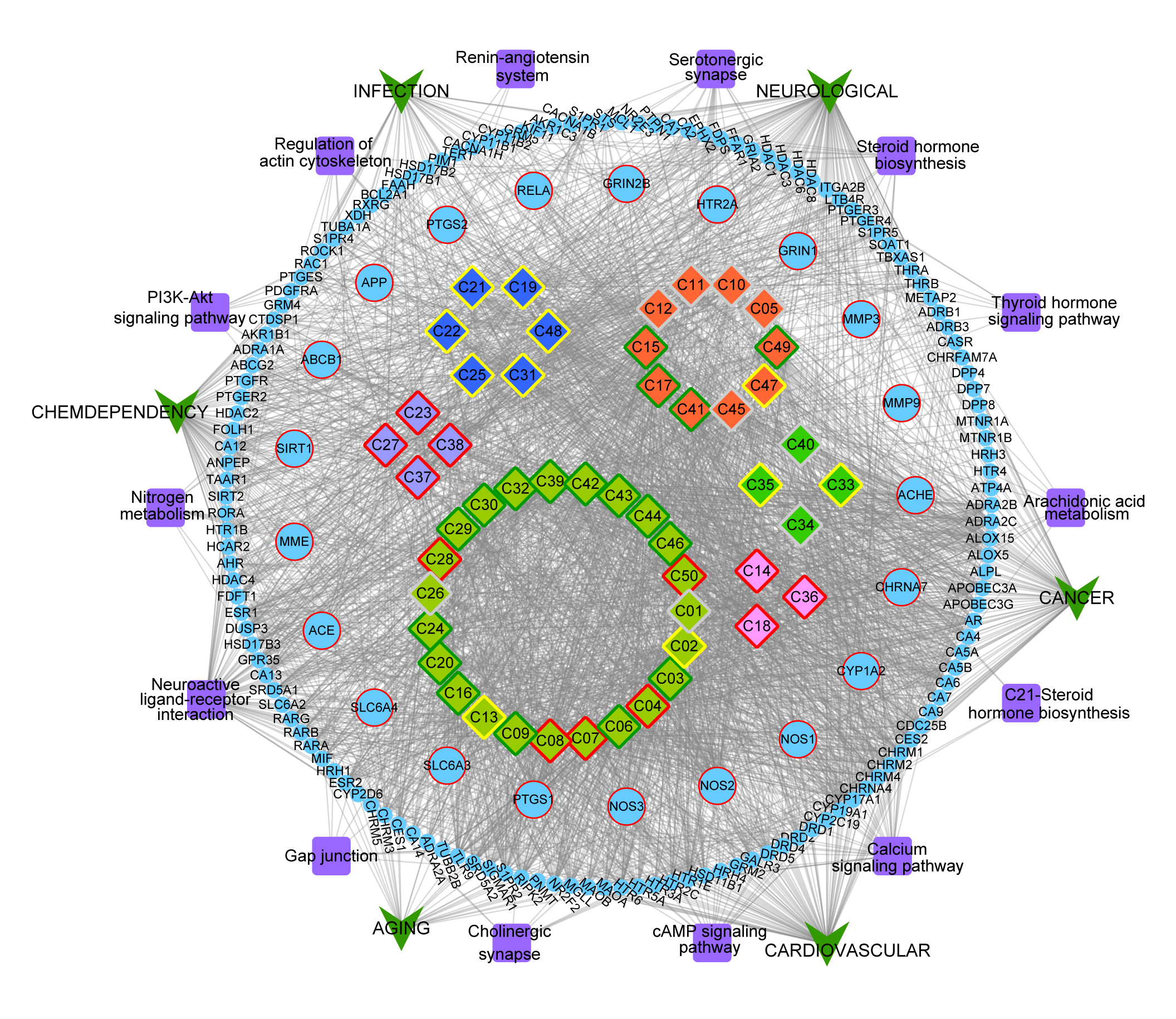

Supplement: Supplementary file 1 [file ijms-21-02071-s001.zip › Supplementary Materials-v6.-revised/Figure S3.tif]

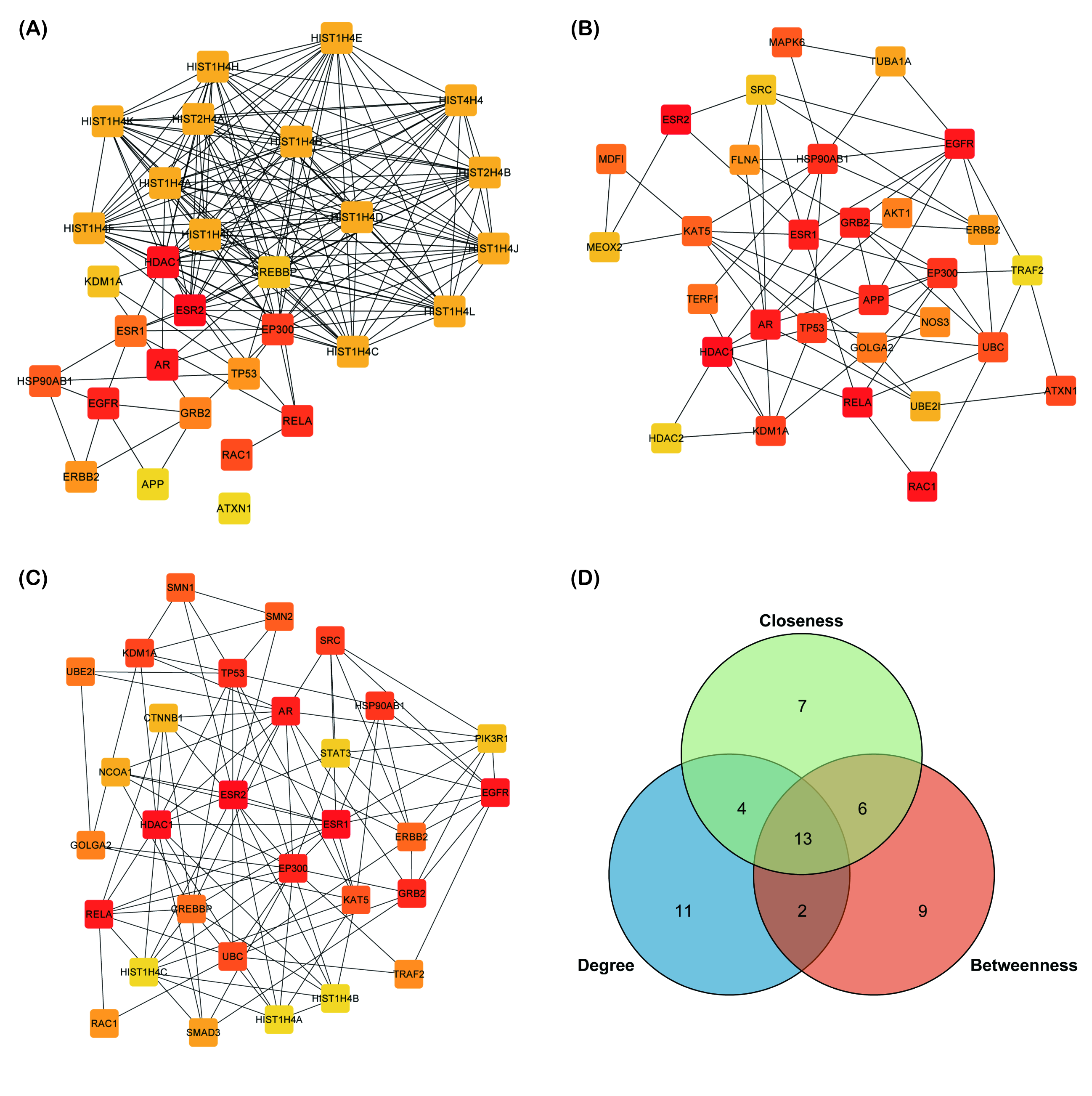

Supplement: Supplementary file 1 [file ijms-21-02071-s001.zip › Supplementary Materials-v6.-revised/Figure S4.tif]

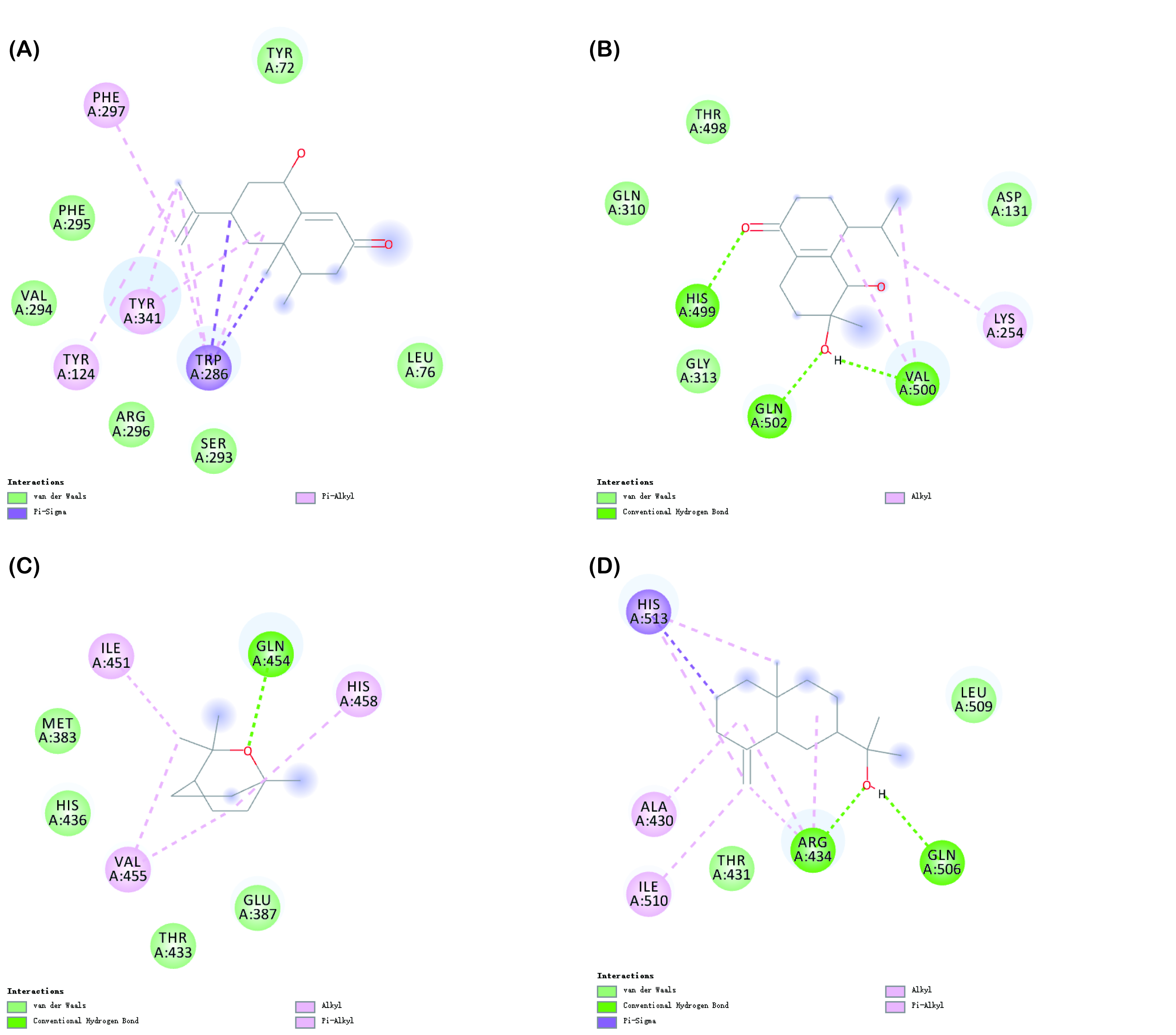

Supplement: Supplementary file 1 [file ijms-21-02071-s001.zip › Supplementary Materials-v6.-revised/Figure S5.tif]
